# Supplementary material for: Original COVID-19 priming regimen impacts the immunogenicity of bivalent BA.1 and BA.5 boosters
Source: Nat Commun. 2024 May 18;15:4224. doi: 10.1038/s41467-024-48414-x (PMC11102539; doi:10.1038/s41467-024-48414-x)
Supplement: Supplementary file 1 — Supplementary Information [file 41467_2024_48414_MOESM1_ESM.pdf]

SUPPLEMENTAL MATERIAL BELONGING TO

Original COVID-19 priming regimen impacts the immunogenicity of bivalent BA.1 and BA.5 boosters

Luca M. Zaeck[1,13], Ngoc H. Tan[2,13], Wim J.R. Rietdijk[2], Daryl Geers[1], Roos S.G. Sablerolles[2], Susanne Bogers[1], Laura L.A. van Dijk[1], Lennert Gommers[1], Leanne P.M. van Leeuwen[1], Sharona Rugebregt[1], Abraham Goorhuis[3,4], Douwe F. Postma[5], Leo G. Visser[6], Virgil A.S.H. Dalm[7,8], Melvin Lafeber[9], Neeltje A. Kootstra[10], Anke L.W. Huckriede[11], Bart L. Haagmans[1], Debbie van Baarle[11,12], Marion P.G. Koopmans[1], SWITCH-ON Research Group*, P. Hugo M. van der Kuy[2,14], Corine H. GeurtsvanKessel[1,14,$], Rory D. de Vries[1,14]

[1] Department of Viroscience, Erasmus University Medical Center, Rotterdam, the Netherlands

[2] Department of Hospital Pharmacy, Erasmus University Medical Center, Rotterdam, the Netherlands

[3] Center of Tropical Medicine and Travel Medicine, Department of Infectious Diseases, Amsterdam University Medical Centers, Amsterdam, the Netherlands

[4] Infection and Immunity, Amsterdam Public Health, University of Amsterdam, Amsterdam, the Netherlands

[5] Department of Internal Medicine and Infectious Diseases, University Medical Center Groningen, Groningen, the Netherlands

[6] Department of Infectious Diseases, Leiden University Medical Center, Leiden, the Netherlands

[7] Department of Internal Medicine, Division of Allergy and Clinical Immunology, Erasmus University Medical Center, Rotterdam, the Netherlands

[8] Department of Immunology, Erasmus University Medical Center, Rotterdam, the Netherlands

[9] Department of Internal Medicine, Erasmus University Medical Center, Rotterdam, the Netherlands

[10] Department of Experimental Immunology, Amsterdam University Medical Centers, Amsterdam Infection and Immunity Institute, University of Amsterdam, Amsterdam, the Netherlands

[11] Department of Medical Microbiology and Infection Prevention, University Medical Center Groningen, University of Groningen, Groningen, the Netherlands

[12] Center for Infectious Disease Control, National Institute for Public Health and the Environment, Bilthoven, the Netherlands

[13] These authors contributed equally: Luca M. Zaeck, Ngoc H. Tan

[14] These authors jointly supervised this work: P. Hugo M. van der Kuy, Corine H. GeurtsvanKessel, Rory D. de Vries

* A list of authors and their affiliations appears at the end of the paper.

[$] corresponding author: Corine H. GeurtsvanKessel, Department of Viroscience, Erasmus University Medical Center, Rotterdam, 3015GD, Netherlands, c.geurtsvankessel@erasmusmc.nl

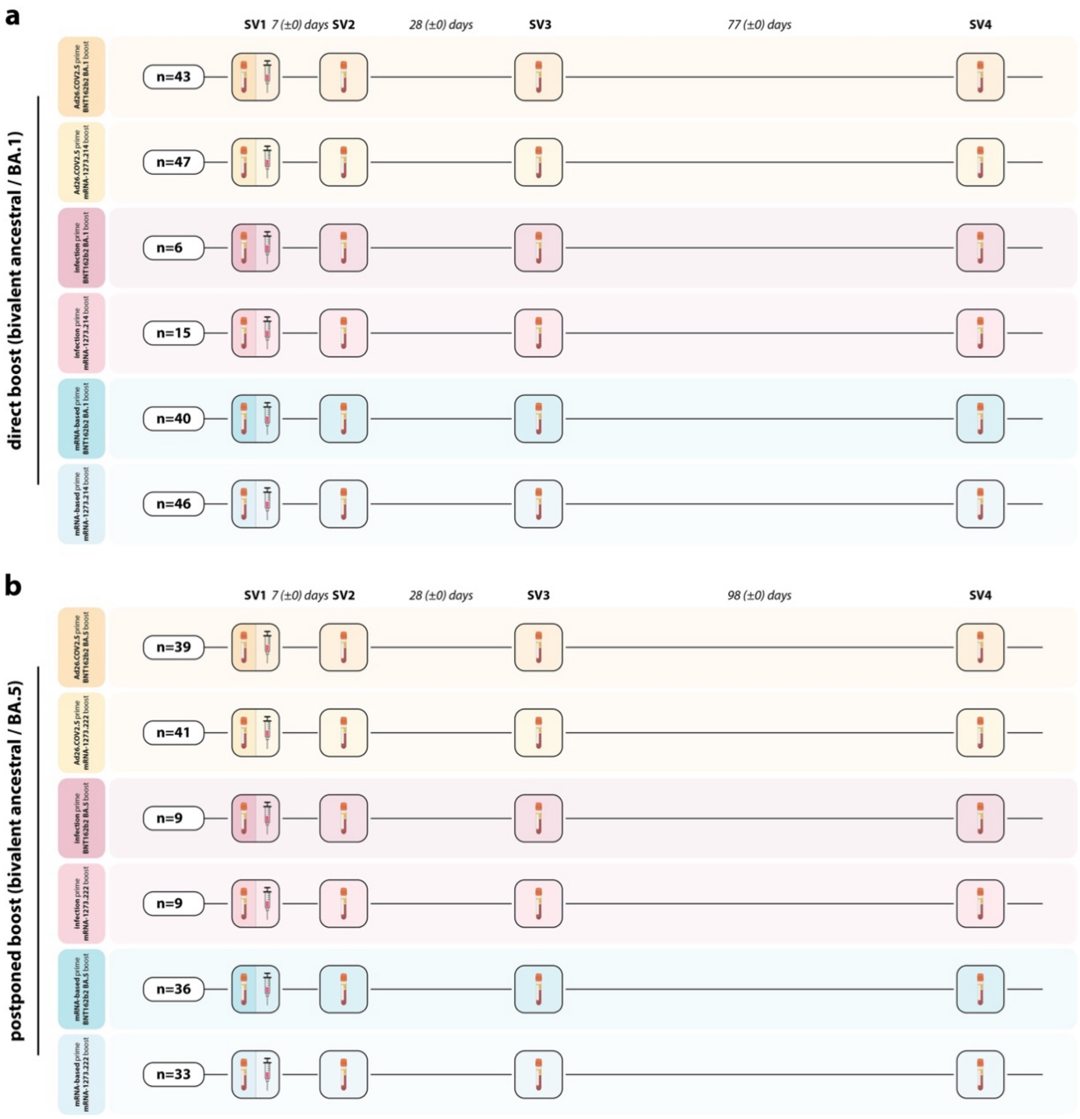

Supplementary Figure S1. SWITCH-ON study design. a,b, Healthcare workers (HCW), who received a priming vaccination regimen with either Ad26.COV2.S (orange and yellow) or an mRNA-based vaccine (dark and light blue), or were infected with SARS-CoV-2 before first vaccination (dark and light red), followed by an additional boost with an mRNA-based vaccine, were vaccinated with either bivalent Omicron BA.1 (**a**, direct boost group) or BA.5 (**b**, postponed boost group). Blood samples were collected at baseline before bivalent vaccination (study visit 1, SV1), at 7 (study visit 2, SV2) and 28 days post-vaccination (study visit 3, SV3), and at approximately 3 months-post vaccination (study visit 4, SV4). The numerical value in brackets indicates the interquartile range (IQR) of days deviating from the intended interval. Created with BioRender.com

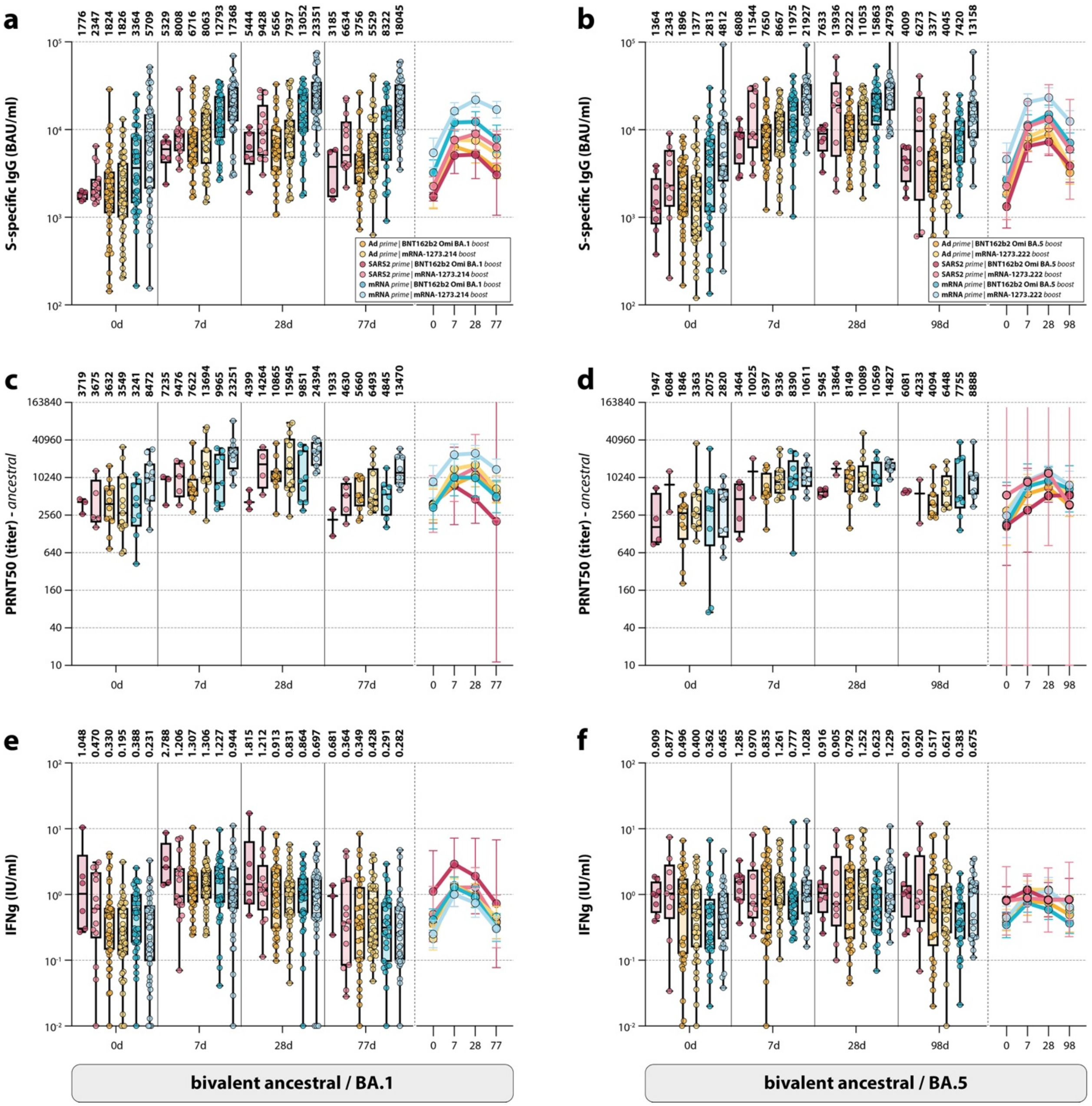

Supplementary Figure S2. Antibody and T-cell responses in subgroups based on the type of first antigen exposure and bivalent booster vaccinations. Detection of (ancestral) spike (S)-specific binding IgG antibodies (**a,b**), ancestral SARS-CoV-2 neutralizing antibodies (**c,d**), and T-cell responses measured by interferon-gamma (IFN-γ) release assay (IGRA) (**e,f**) based on the different combinations of original priming regimen after Omicron BA.1 (**a,c,e**) or BA.5 (**b,d,f**) bivalent booster vaccination at baseline, and 7 days, 28 days, and 3 months post-boost.

Colors indicate the specific prime-boost regimen (orange = Ad26.COV2.S prime, BNT162b2 Omicron BA.1 or BA.5 boost; yellow = Ad26.COV2.S prime, mRNA-1273.214 or mRNA-1273.222 boost; dark red = SARS-CoV-2 infection prime, BNT162b2 Omicron BA.1 or BA.5 boost; light red = SARS-CoV-2 infection prime, mRNA-1273.214 or mRNA-1273.222 boost; dark blue = mRNA-based prime, BNT162b2 Omicron BA.1 or BA.5 boost; light blue = mRNA-based prime, mRNA-1273.214 or mRNA-1273.222 boost). Data are shown in box-and-whisker plots, with the horizontal lines indicating the median, the bounds of the boxes indicating the interquartile range (IQR), and the whiskers indicating the range. Bold numbers above the plots represent the respective geometric mean (titer) per timepoint. The line graphs next to each panel depict a time course of the respective geometric mean values with 95% confidence intervals. The number of biologically independent samples (serum or whole blood) used per assay are shown in Supplementary Table S4.

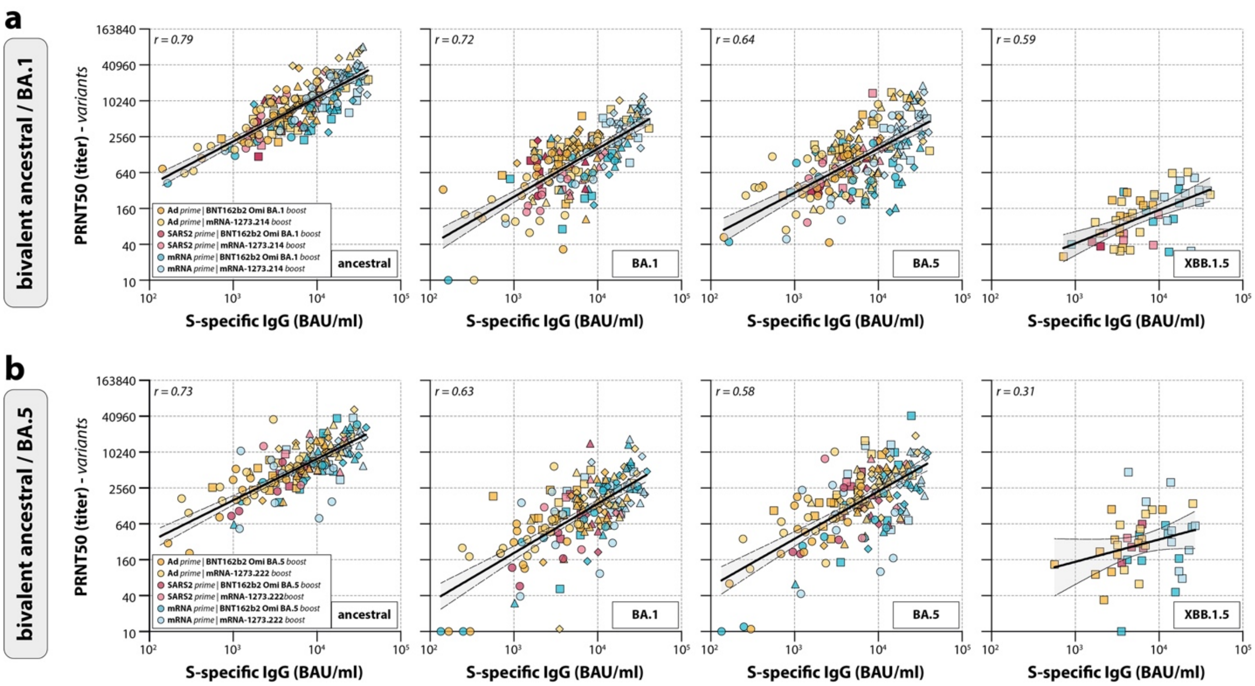

Supplementary Figure S3. Correlations between ancestral SARS-CoV-2 and variant-specific neutralizing antibodies. a,b, Correlations between neutralizing antibodies against ancestral SARS-CoV-2 and the Omicron BA.1, BA.5, and XBB.1.5 variants after Omicron BA.1 (**a**) or BA.5 (**b**) bivalent vaccination at 3 months post-boost. Colors indicate the specific prime-boost regimen (orange = Ad26.COV2.S prime, BNT162b2 Omicron BA.1 or BA.5 boost; yellow = Ad26.COV2.S prime, mRNA-1273.214 or mRNA-1273.222 boost; dark red = SARS-CoV-2 infection prime, BNT162b2 Omicron BA.1 or BA.5 boost; light red = SARS-CoV-2 infection prime, mRNA-1273.214 or mRNA-1273.222 boost; dark blue = mRNA-based prime, BNT162b2 Omicron BA.1 or BA.5 boost; light blue = mRNA-based prime, mRNA-1273.214 or mRNA-1273.222 boost). Correlations were evaluated by Spearman's r. The number of biologically independent serum samples used per assay are shown in Supplementary Table S4.

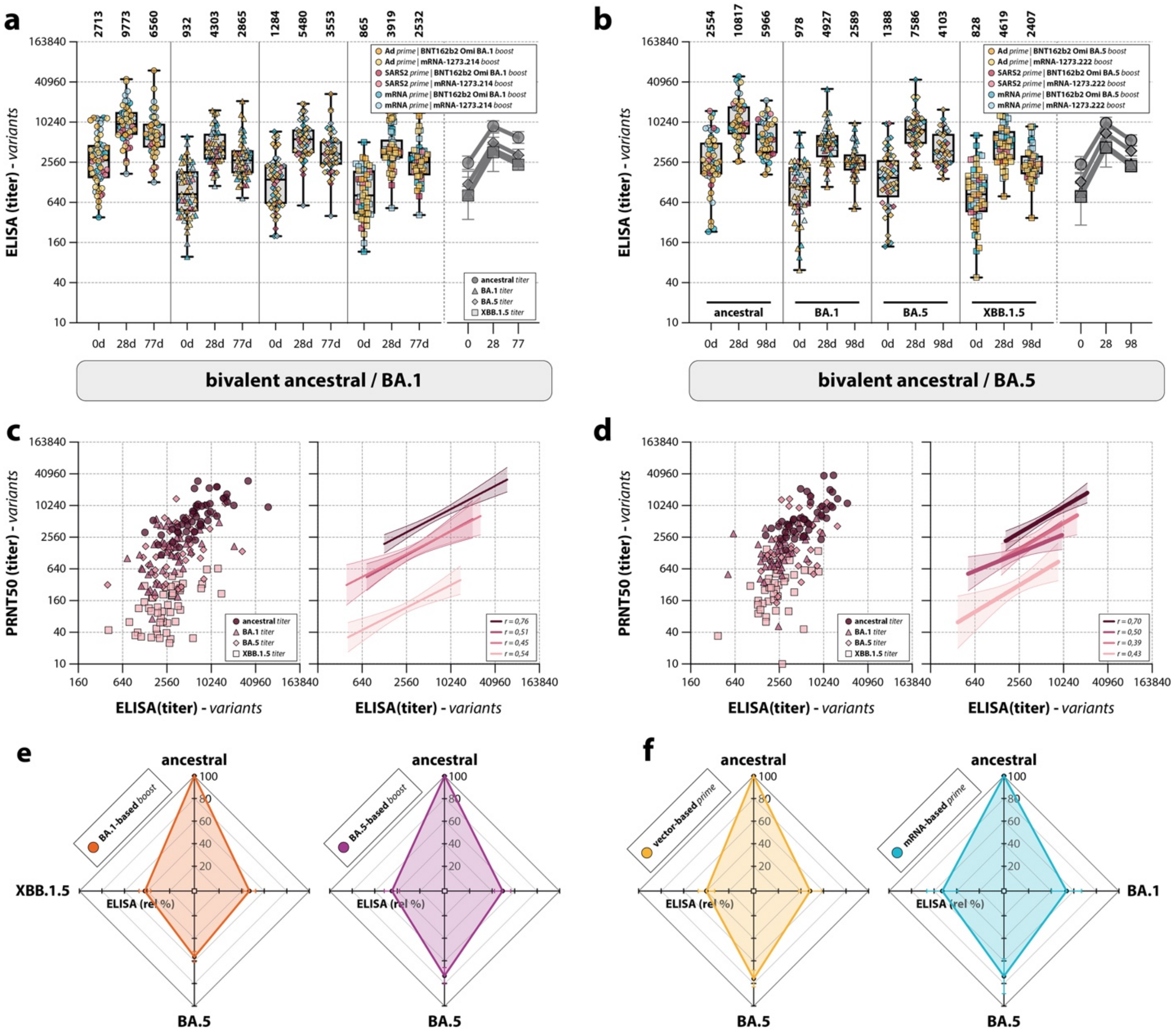

Supplementary Figure S4. Breadth of the binding antibody response after bivalent booster vaccination. a,b, Detection of binding antibodies targeting ancestral SARS-CoV-2 and Omicron BA.1, BA.5, and XBB.1.5 variants after Omicron BA.1 (**a**) or BA.5 (**b**) bivalent booster vaccination at baseline, and 7 days, 28 days, and 3 months post-boost. Colors indicate the specific prime-boost regimen (orange = Ad26.COV2.S prime, BNT162b2 Omicron BA.1 or BA.5 boost; yellow = Ad26.COV2.S prime, mRNA-1273.214 or mRNA-1273.222 boost; dark red = SARS-CoV-2 infection prime, BNT162b2 Omicron BA.1 or BA.5 boost; light red = SARS-CoV-2 infection prime, mRNA-1273.214 or mRNA-1273.222 boost; dark blue = mRNA-based prime, BNT162b2 Omicron BA.1 or BA.5 boost; light blue = mRNA-based prime, mRNA-1273.214 or mRNA-1273.222 boost).

c-d, Correlations between binding and neutralizing antibody titers against ancestral SARS-CoV-2 (circles) and the Omicron BA.1 (triangles), BA.5 (diamonds) and XBB.1.5 (squares) variants after Omicron BA.1 (**c**) or BA.5 (**d**) bivalent vaccination at 3 months post-boost. Correlations were evaluated by Spearman's r. **e,f**, Radar plots depicting the variant-specific binding antibody titers relative to ancestral SARS-CoV-2 binding (set to 100%) after vaccination with bivalent Omicron BA.1 or BA.5. The plots are grouped either by the administered Omicron BA.1 (orange) or BA.5 (purple) bivalent booster vaccination (**e**) or the original priming regimen (vector-based = yellow; mRNA-based = blue) after Omicron BA.5 bivalent vaccination (**f**). Data in panels **a,b** are shown in box-and-whisker plots, with the horizontal lines indicating the median, the bounds of the boxes indicating the interquartile range (IQR), and the whiskers indicating the range. Bold numbers above the plots represent the respective geometric mean (titer) per timepoint. The line graphs next to each panel depict a time course of the respective geometric mean values with 95% confidence intervals. The number of biologically independent serum samples used per assay are shown in Supplementary Table S4.

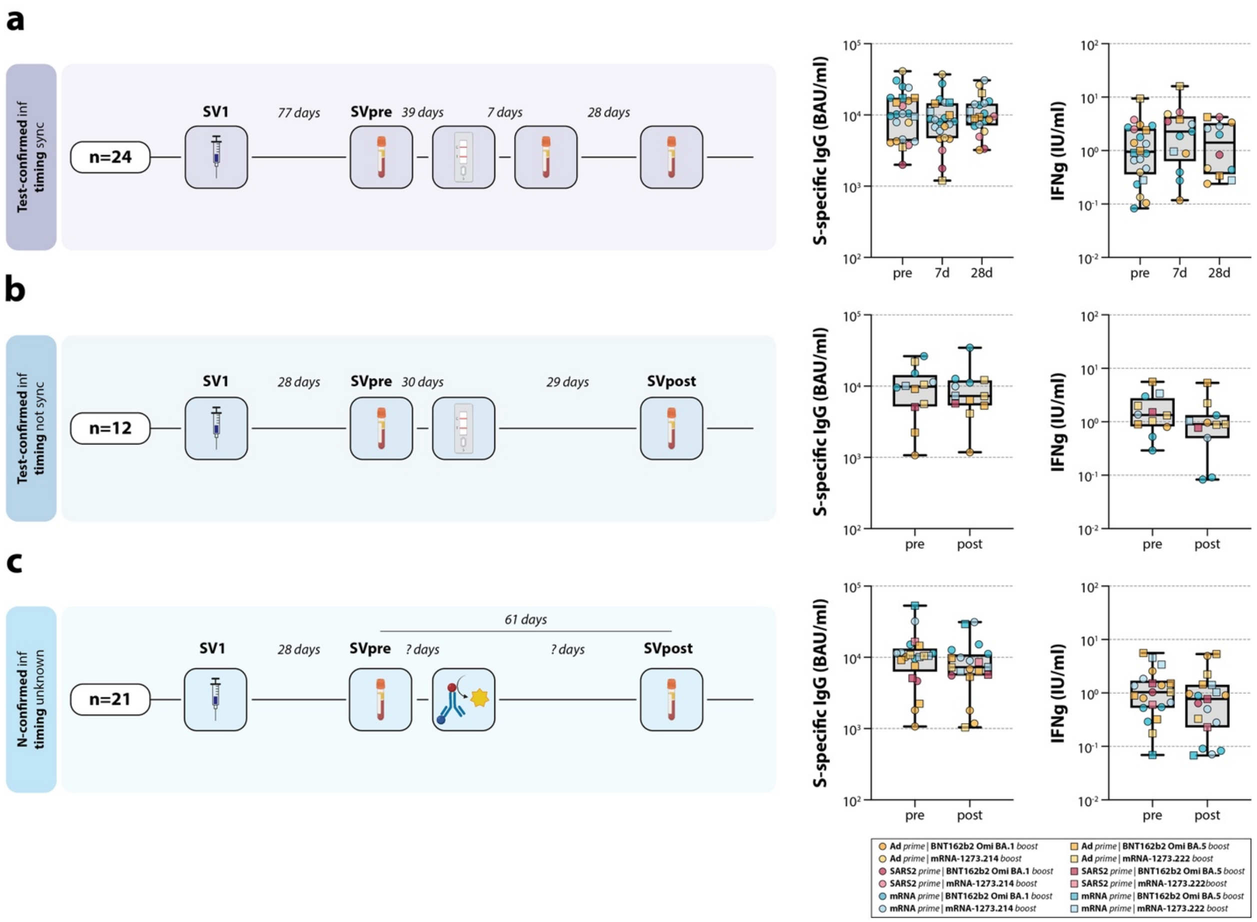

Supplementary Figure S5. Overview of post-vaccination breakthrough infections during the SWITCH-ON trial. a-c, Sampling procedure (left), and detection of S-specific IgG antibodies (middle) and T-cell responses measured by interferon-gamma (IFN-γ) release assay (IGRA; right) for participants who had a breakthrough infection after Omicron BA.1 or BA.5 bivalent booster vaccination (study visit 1, SV1). Samples were either collected analogous to the pre-vaccination breakthrough infection group depicted in **Figure 6** at 7 and 28 days after testing positive (**a**, n = 24), or outside of this regimen as a pre- and post-infection sample (**b**, n = 12; **c**, n = 21). Infections were either confirmed by positive test result (**a,b**), or via detection of nucleocapsid-specific antibodies (N-ELISA) (**c**). Depictions of sampling procedures on the left-hand side were created with BioRender.com.

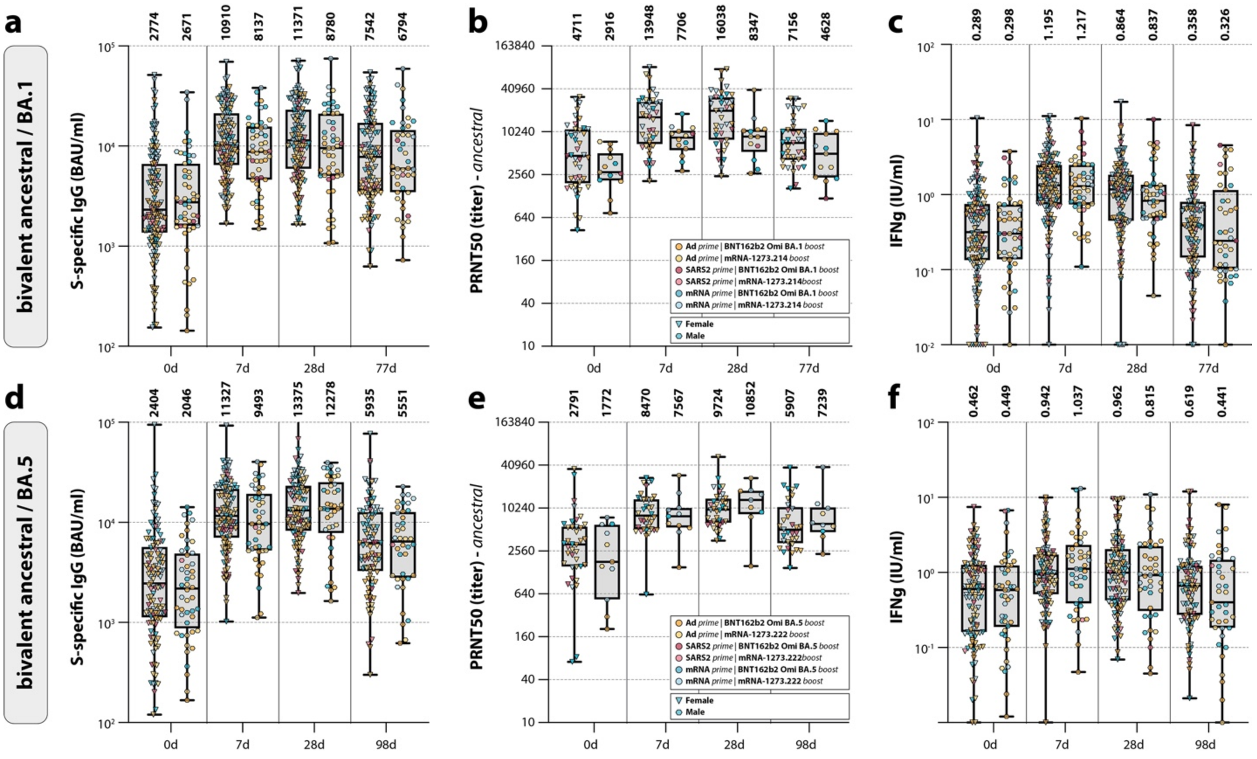

Supplementary Figure S6. Antibody and T-cell responses after bivalent booster vaccination grouped by sex. a-f, Detection of (ancestral) spike (S)-specific binding IgG antibodies (a,d), ancestral SARS-CoV-2 neutralizing antibodies (b,e), and T-cell responses measured by interferon-gamma (IFN-γ) release assay (IGRA) (c,f) after Omicron BA.1 (a-c) or BA.5 (d-f) bivalent booster vaccination at baseline, and 7 days, 28 days, and 3 months post-boost, grouped by sex (female = triangle; male = circle). Colors indicate the specific prime-boost regimen (orange = Ad26.COV2.S prime, BNT162b2 Omicron BA.1 or BA.5 boost; yellow = Ad26.COV2.S prime, mRNA-1273.214 or mRNA-1273.222 boost; dark red = SARS-CoV-2 infection prime, BNT162b2 Omicron BA.1 or BA.5 boost; light red = SARS-CoV-2 infection prime, mRNA-1273.214 or mRNA-1273.222 boost; dark blue = mRNA-based prime, BNT162b2 Omicron BA.1 or BA.5 boost; light blue = mRNA-based prime, mRNA-1273.214 or mRNA-1273.222 boost). Data are shown in box-and-whisker plots, with the horizontal lines indicating the median, the bounds of the boxes indicating the interquartile range (IQR), and the whiskers indicating the range. Bold numbers above the plots represent the respective geometric mean (titer) per timepoint.

Supplementary Table 1. Baseline characteristics of trial participants in direct boost group

		Direct boost group						
		Total<br>n = 197	Ad26.COV.S / BNT162b2.BA.1<br>n = 43	mRNA / BNT162b2.BA.1<br>n = 40	SARS-CoV-2 / BNT162b2.BA.1<br>n = 6	Ad26.COV.S / mRNA-1273.214<br>n = 47	mRNA / mRNA-1273.214<br>n = 46	SARS-CoV-2 / mRNA-1273.214<br>n = 15
	**Female**	149 (76%)	29 (67%)	31 (78%)	3 (50%)	29 (62%)	40 (87%)	14 (93%)
	**Male**	48 (24%)	14 (33%)	9 (23%)	3 (50%)	18 (38%)	6 (13%)	1 (7%)
	**Age**	47.0 (36.0-53.0)	36.0 (28.0-40.5)	35.0 (28.0-40.0)	32.0 (28.0-36.8)	53.0 (50.0-55.0)	53.5 (50.0-57.0)	52.0 (50.5-54.0)
	**BMI**	24.4 (22.6-27.3)	23.9 (22.4-25.6)	23.8 (21.7-26.3)	24.1 (21.7-29.2)	25.3 (23.0-27.8)	25.2 (23.2-27.8)	23.9 (23.0-27.1)
**Ancestry**	**African**	2 (1%)	0 (0%)	1 (3%)	0 (0%)	1 (2%)	0 (0%)	0 (0%)
	**Asian**	6 (3%)	0 (0%)	3 (8%)	0 (0%)	0 (0%)	3 (7%)	0 (0%)
	**European**	183 (93%)	41 (95%)	35 (88%)	6 (100%)	46 (98%)	41 (89%)	14 (93%)
	**North-American**	1 (1%)	0 (0%)	1 (3%)	0 (0%)	0 (0%)	0 (0%)	0 (0%)
	**South-American**	0 (0%)	0 (0%)	0 (0%)	0 (0%)	0 (0%)	0 (0%)	0 (0%)
	**Other**	5 (3%)	2 (5%)	0 (0%)	0 (0%)	0 (0%)	2 (4%)	1 (7%)
**Occupation in hospital**	**Administrative/policy maker**	29 (15%)	7 (16%)	3 (8%)	0 (0%)	11 (23%)	7 (15%)	1 (7%)
	**Medical doctor**	15 (8%)	2 (5%)	4 (10%)	0 (0%)	0 (0%)	7 (15%)	2 (13%)
	**Facility services**	3 (2%)	1 (2%)	1 (3%)	0 (0%)	1 (2%)	0 (0%)	0 (0%)
	**Management**	25 (13%)	5 (12%)	2 (5%)	1 (17%)	8 (17%)	6 (13%)	3 (20%)
	**Supportive staff clinic/emergency department**	2 (1%)	1 (2%)	0 (0%)	0 (0%)	0 (0%)	1 (2%)	0 (0%)
	**Supportive staff outpatient clinic**	2 (1%)	0 (0%)	0 (0%)	0 (0%)	0 (0%)	1 (2%)	1 (7%)
	**Researcher**	45 (23%)	16 (37%)	13 (33%)	2 (33%)	6 (13%)	8 (17%)	0 (0%)
	**Nurse**	11 (6%)	0 (0%)	3 (8%)	1 (17%)	0 (0%)	4 (9%)	3 (20%)
	**Other**	65 (33%)	11 (26%)	14 (35%)	2 (33%)	21 (45%)	12 (26%)	5 (33%)
**Comorbidities**	**Cardiovascular diseases**	2 (1%)	0 (0%)	0 (0%)	0 (0%)	0 (0%)	2 (4%)	0 (0%)
	**Pulmonary diseases**	10 (5%)	1 (2%)	2 (5%)	0 (0%)	2 (4%)	1 (2%)	4 (27%)
	**Diabetes mellitus**	3 (2%)	0 (0%)	0 (0%)	1 (17%)	2 (4%)	0 (0%)	0 (0%)
	**Liver diseases**	2 (1%)	0 (0%)	0 (0%)	0 (0%)	0 (0%)	2 (4%)	0 (0%)
	**Kidney diseases**	3 (2%)	1 (2%)	1 (3%)	0 (0%)	0 (0%)	1 (2%)	0 (0%)
**Nucleocapsid on study visit 1 (vaccination day)**	**Negative**	161 (82%)	34 (79%)	35 (88%)	6 (100%)	44 (94%)	35 (76%)	7 (47%)
	**Positive**	36 (18%)	9 (21%)	5 (13%)	0 (0%)	3 (6%)	11 (24%)	8 (53%)
**Original center**	**Amsterdam University Medical Center**	22 (11%)	5 (12%)	6 (15%)	3 (50%)	3 (6%)	4 (9%)	1 (7%)
	**Erasmus Medical Center Rotterdam**	135 (69%)	24 (56%)	32 (80%)	3 (50%)	22 (47%)	40 (87%)	14 (93%)
	**Leiden University Medical Center**	16 (8%)	8 (19%)	0 (0%)	0 (0%)	8 (17%)	0 (0%)	0 (0%)
	**University Medical Center Groningen**	24 (12%)	6 (14%)	2 (5%)	0 (0%)	14 (30%)	2 (4%)	0 (0%)
**Median time**	**Between study 1 and 2 (day 0 and 7)**	7.0 (7.0-7.0)	7.0 (7.0-7.0)	7.0 (7.0-7.0)	7.0 (7.0-7.0)	7.0 (7.0-7.0)	7.0 (7.0-7.0)	7.0 (7.0-7.0)
	**Between study 1 and 3 (day 0 and 28)**	28.0 (28.0-28.0)	28.0 (28.0-28.0)	28.0 (28.0-28.0)	28.0 (28.0-28.0)	28.0 (28.0-28.0)	28.0 (28.0-28.0)	28.0 (28.0-28.0)
	**Between study 1 and 4 (day 0 and 90)**	77.0 (77.0-77.0)	77.0 (77.0-77.0)	77.0 (77.0-77.0)	77.0 (77.0-77.0)	77.0 (77.0-77.0)	77.0 (77.0-77.0)	77.0 (77.0-77.0)
	**Between last booster and bivalent booster**	298.0 (266.0-310.0)	266.0 (259.0-308.0)	303.0 (297.0-310.0)	304.0 (298.3-308.3)	266.0 (261.5-268.0)	307.0 (303.0-310.0)	305.0 (301.5-310.5)

Note: for categorical variables we present numbers (percentages), whereas continuous variables we present median (interquartile range).

Supplementary Table 2. Baseline characteristics of trial participants in postponed boost group

		Postponed boost group						
		**Total** n = 167	**Ad26.COV.S / BNT162b2.BA.5** n = 39	**mRNA / BNT162b2.BA.5** n = 36	**SARS-CoV-2 / BNT162b2.BA.5** n = 9	**Ad26.COV.S / mRNA-1273.222** n = 41	**mRNA / mRNA-1273.222** n = 33	**SARS-CoV-2 / mRNA-1273.222** n = 9
	**Female**	119 (71%)	24 (62%)	25 (69%)	9 (100%)	30 (73%)	23	8 (89%)
	**Male**	48 (29%)	15 (38%)	11 (31%)	0 (0%)	11 (27%)	10	1 (11%)
	**Age**	47.0 (37.0-53.0)	47.0 (38.0-54.0)	46.5 (37.0-52.3)	50.0 (49.0-51.0)	45.0 (36.0-54.0)	45.0 (37.0-53.0)	48.0 (43.0-52.0)
	**BMI**	24.7 (22.2-27.7)	24.7 (22.4-27.2)	25.1 (22.1-28.6)	28.4 (25.8-31.6)	24.1 (21.4-25.8)	25.3 (22.2-28.7)	24.0 (23.1-24.1)
**Ancestry**	**African**	0 (0%)	0 (0%)	0 (0%)	0 (0%)	0 (0%)	0 (0%)	0 (0%)
	**Asian**	4 (2%)	0 (0%)	1 (3%)	0 (0%)	1 (2%)	2 (6%)	0 (0%)
	**European**	153 (92%)	38 (97%)	32 (89%)	9 (100%)	37 (90%)	28 (85%)	9 (100%)
	**North-American**	0 (0%)	0 (0%)	0 (0%)	0 (0%)	0 (0%)	0 (0%)	0 (0%)
	**South-American**	3 (2%)	0 (0%)	1 (3%)	0 (0%)	1 (2%)	1 (3%)	0 (0%)
	**Other**	7 (4%)	1 (3%)	2 (6%)	0 (0%)	2 (5%)	2 (6%)	0 (0%)
**Occupation in hospital**	**Administrative/policy maker**	22 (13%)	4 (10%)	2 (6%)	2 (22%)	6 (15%)	6 (18%)	2 (22%)
	**Medical doctor**	14 (8%)	1 (3%)	6 (17%)	1 (11%)	0 (0%)	6 (18%)	0 (0%)
	**Facility services**	4 (2%)	2 (5%)	0 (0%)	0 (0%)	2 (5%)	0 (0%)	0 (0%)
	**Management**	13 (8%)	2 (5%)	1 (3%)	0 (0%)	6 (15%)	3 (9%)	1 (11%)
	**Supportive staff clinic/emergency department**	3 (2%)	0 (0%)	1 (3%)	1 (11%)	0 (0%)	1 (3%)	0 (0%)
	**Supportive staff outpatient clinic**	1 (1%)	0 (0%)	1 (3%)	0 (0%)	0 (0%)	0 (0%)	0 (0%)
	**Researcher**	30 (18%)	10 (26%)	3 (8%)	2 (22%)	10 (24%)	3 (9%)	2 (22%)
	**Nurse**	13 (8%)	1 (3%)	7 (19%)	1 (11%)	0 (0%)	3 (9%)	1 (11%)
	**Other**	67 (40%)	19 (49%)	15 (42%)	2 (22%)	17 (41%)	11 (33%)	3 (33%)
**Comorbidities**	**Cardiovascular diseases**	4 (2%)	0 (0%)	1 (3%)	0 (0%)	0 (0%)	2 (6%)	1 (11%)
	**Pulmonary diseases**	6 (4%)	4 (10%)	1 (3%)	0 (0%)	0 (0%)	1 (3%)	0 (0%)
	**Diabetes mellitus**	2 (1%)	0 (0%)	1 (3%)	0 (0%)	0 (0%)	0 (0%)	1 (11%)
	**Liver diseases**	2 (1%)	1 (3%)	0 (0%)	0 (0%)	1 (2%)	0 (0%)	0 (0%)
	**Kidney diseases**	0 (0%)	0 (0%)	0 (0%)	0 (0%)	0 (0%)	0 (0%)	0 (0%)
**Nucleocapsid on study visit 1 (vaccination day)**	**Negative**	126 (75%)	30 (77%)	27 (75%)	6 (67%)	32 (78%)	26 (79%)	5 (56%)
	**Positive**	40 (24%)	9 (23%)	8 (22%)	3 (33%)	9 (22%)	7 (21%)	4 (44%)
**Original center**	**Amsterdam University Medical Center**	21 (13%)	5 (13%)	5 (14%)	1 (11%)	6 (15%)	3 (9%)	1 (11%)
	**Erasmus Medical Center Rotterdam**	114 (68%)	22 (56%)	29 (81%)	8 (89%)	19 (46%)	29 (88%)	7 (78%)
	**Leiden University Medical Center**	13 (8%)	6 (15%)	0 (0%)	0 (0%)	7 (17%)	0 (0%)	0 (0%)
	**University Medical Center Groningen**	18 (11%)	6 (15%)	1 (3%)	0 (0%)	9 (22%)	1 (3%)	1 (11%)
**Median time**	**Between study 1 and 2 (day 0 and 7)**	7.0 (7.0-7.0)	7.0 (7.0-7.0)	7.0 (7.0-7.0)	7.0 (7.0-7.0)	7.0 (7.0-7.0)	7.0 (7.0-7.0)	7.0 (7.0-7.0)
	**Between study 1 and 3 (day 0 and 28)**	28.0 (28.0-28.0)	28.0 (28.0-28.0)	28.0 (28.0-28.0)	28.0 (28.0-28.0)	28.0 (28.0-28.0)	28.0 (28.0-28.0)	28.0 (28.0-28.0)
	**Between study 1 and 4 (day 0 and 90)**	98.0 (98.0-98.0)	98.0 (98.0-98.0)	98.0 (98.0-98.0)	98.0 (98.0-98.0)	98.0 (98.0-98.0)	98.0 (98.0-98.0)	98.0 (98.0-98.0)
	**Between last booster and bivalent booster**	366.0 (336.0-380.0)	336.0 (329.5-364.0)	380.0 (370.0-382.0)	376.0 (366.0-380.0)	337.0 (333.0-364.0)	376.0 (366.0-381.0)	376.0 (373.0-380.0)

Note: for categorical variables we present numbers (percentages). whereas continuous variables we present median (interquartile range).
Note: one participant refused to disclose their weight, therefore the BMI was excluded.

Supplementary Table S3. Baseline table on the number of antigen exposures per original priming regimen and bivalent booster vaccination.

	Vaccines prior to bivalent boost			Infections prior to bivalent boost		
	**2**	**3**	**4**	**0**	**1**	**2**
**mRNA | BNT162b2 BA.1**	0 (0%)	40 (100%)	0 (0%)	11 (28%)	28 (70%)	1 (3%)
**mRNA | mRNA-1273.214**	0 (0%)	43 (93%)	3 (7%)	14 (30%)	32 (70%)	0 (0%)
**vector | BNT162b2 BA.1**	15 (35%)	28 (65%)	0 (0%)	12 (28%)	29 (67%)	2 (5%)
**vector | mRNA-1273.214**	12 (26%)	35 (74%)	0 (0%)	18 (38%)	29 (61%)	0 (0%)
**mRNA | BNT162b2 BA.5**	0 (0%)	35 (97%)	1 (3%)	10 (28%)	25 (69%)	1 (3%)
**mRNA | mRNA-1273.222**	0 (0%)	31 (94%)	2 (6%)	11 (33%)	22 (67%)	0 (0%)
**vector | BNT162b2 BA.5**	14 (36%)	25 (64%)	0 (0%)	11 (28%)	27 (69%)	1 (3%)
**vector | mRNA-1273.222**	15 (37%)	26 (63%)	0 (0%)	15 (37%)	26 (63%)	0 (0%)

Supplementary Table S4. Data availability for both groups.

	Direct boost group (n=197)	Postponed boost group (n=167)
	**Available data (%)**	**Available data (%)**
**Liaison - Study visit 1**	197 (100%)	166 (99%)
**Liaison - Study visit 2**	191 (97%)	161 (96%)
**Liaison - Study visit 3**	181 (92%)	151 (90%)
**Liaison - Study visit 4**	168 (85%)	137 (82%)
**Nucleocapsid - Study visit 1**	197 (100%)	166 (99%)
**Nucleocapsid - Study visit 4**	171 (87%)	144 (86%)
**IGRA Ag2 - Study visit 1**	187 (95%)	159 (95%)
**IGRA Ag2 - Study visit 2**	185 (94%)	154 (92%)
**IGRA Ag2 - Study visit 3**	173 (88%)	143 (86%)
**IGRA Ag2 - Study visit 4**	157 (80%)	132 (79%)
**PRNT50 ancestral - Study visit 1**	55 (28%)	49 (29%)
**PRNT50 ancestral - Study visit 2**	55 (28%)	49 (29%)
**PRNT50 ancestral - Study visit 3**	52 (26%)	45 (27%)
**PRNT50 ancestral - Study visit 4**	50 (25%)	43 (26%)
**PRNT50 BA.1 - Study visit 1**	54 (27%)	49 (29%)
**PRNT50 BA.1 - Study visit 2**	55 (28%)	49 (29%)
**PRNT50 BA.1 - Study visit 3**	52 (26%)	45 (27%)
**PRNT50 BA.1 - Study visit 4**	50 (25%)	43 (26%)
**PRNT50 BA.5 - Study visit 1**	55 (28%)	49 (29%)
**PRNT50 BA.5 - Study visit 2**	55 (28%)	49 (29%)
**PRNT50 BA.5 - Study visit 3**	52 (26%)	45 (27%)
**PRNT50 BA.5 - Study visit 4**	50 (25%)	43 (26%)
**PRNT50 XBB.1.5 - Study visit 1**	0 (0%)	0 (0%)
**PRNT50 XBB.1.5 - Study visit 2**	0 (0%)	0 (0%)
**PRNT50 XBB.1.5 - Study visit 3**	0 (0%)	0 (0%)
**PRNT50 XBB.1.5 - Study visit 4**	50 (25%)	43 (26%)
**ELISA ancestral - Study visit 1**	55 (28%)	49 (29%)
**ELISA ancestral - Study visit 2**	0 (0%)	0 (0%)
**ELISA ancestral - Study visit 3**	51 (26%)	48 (29%)
**ELISA ancestral - Study visit 4**	50 (25%)	43 (26%)
**ELISA BA.1 - Study visit 1**	55 (28%)	49 (29%)
**ELISA BA.1 - Study visit 2**	0 (0%)	0 (0%)
**ELISA BA.1 - Study visit 3**	51 (26%)	48 (29%)
**ELISA BA.1 - Study visit 4**	50 (25%)	43 (26%)
**ELISA BA.5 - Study visit 1**	55 (28%)	49 (29%)
**ELISA BA.5 - Study visit 2**	0 (0%)	0 (0%)
**ELISA BA.5 - Study visit 3**	51 (26%)	48 (29%)
**ELISA BA.5 - Study visit 4**	50 (25%)	43 (26%)
**ELISA XBB.1.5 - Study visit 1**	55 (28%)	49 (29%)
**ELISA XBB.1.5 - Study visit 2**	0 (0%)	0 (0%)
**ELISA XBB.1.5 - Study visit 3**	51 (26%)	48 (29%)
**ELISA XBB.1.5 - Study visit 4**	50 (25%)	43 (26%)
