# Supplementary material for: Original COVID-19 priming regimen impacts the immunogenicity of bivalent BA.1 and BA.5 boosters
Source: Nat Commun. 2024 May 18;15:4224. doi: 10.1038/s41467-024-48414-x (PMC11102539; doi:10.1038/s41467-024-48414-x)
Supplement: Supplementary file 3 — Reporting Summary [file 41467_2024_48414_MOESM3_ESM.pdf]

nature portfolio

Corresponding author(s): Corine H. GeurtsvanKessel

Last updated by author(s): Apr 18, 2024

Reporting Summary

Nature Portfolio wishes to improve the reproducibility of the work that we publish. This form provides structure for consistency and transparency in reporting. For further information on Nature Portfolio policies, see our Editorial Policies and the Editorial Policy Checklist.

Statistics

For all statistical analyses, confirm that the following items are present in the figure legend, table legend, main text, or Methods section.

n/a	Confirmed	
☐	☒	The exact sample size (*n*) for each experimental group/condition, given as a discrete number and unit of measurement
☐	☒	A statement on whether measurements were taken from distinct samples or whether the same sample was measured repeatedly
☒	☐	The statistical test(s) used AND whether they are one- or two-sided<br>*Only common tests should be described solely by name; describe more complex techniques in the Methods section.*
☒	☐	A description of all covariates tested
☐	☒	A description of any assumptions or corrections, such as tests of normality and adjustment for multiple comparisons
☐	☒	A full description of the statistical parameters including central tendency (e.g. means) or other basic estimates (e.g. regression coefficient) AND variation (e.g. standard deviation) or associated estimates of uncertainty (e.g. confidence intervals)
☐	☒	For null hypothesis testing, the test statistic (e.g. *F*, *t*, *r*) with confidence intervals, effect sizes, degrees of freedom and *P* value noted<br>*Give P values as exact values whenever suitable.*
☒	☐	For Bayesian analysis, information on the choice of priors and Markov chain Monte Carlo settings
☒	☐	For hierarchical and complex designs, identification of the appropriate level for tests and full reporting of outcomes
☐	☒	Estimates of effect sizes (e.g. Cohen's *d*, Pearson's *r*), indicating how they were calculated

Our web collection on statistics for biologists contains articles on many of the points above.

Software and code

Policy information about availability of computer code

Data collection	No software was used, no code was generated.
Data analysis	Statistical analyses were performed with GraphPad Prism v10.2.1. Figures were prepared with GraphPad Prism v10.2.1 and Adobe Illustrator 2024.

For manuscripts utilizing custom algorithms or software that are central to the research but not yet described in published literature, software must be made available to editors and reviewers. We strongly encourage code deposition in a community repository (e.g. GitHub). See the Nature Portfolio guidelines for submitting code & software for further information.

Data

Policy information about availability of data

All manuscripts must include a data availability statement. This statement should provide the following information, where applicable:

- Accession codes, unique identifiers, or web links for publicly available datasets
- A description of any restrictions on data availability
- For clinical datasets or third party data, please ensure that the statement adheres to our policy

The data generated in this study are provided in the Source Data file. Materials and samples are available upon reasonable request from the corresponding author and will be released via a material transfer agreement. The SARS-CoV-2 virus stocks are available through the European Virus Archive Global.

Accession codes of the viruses used in this manuscript:
ancestral (D614G; GISAID: hCov-19/Netherlands/ZH-EMC-2498)
Omicron BA.1 (GISAID: hCoV-19/Netherlands/LI-SQD-01032/2022)
Omicron BA.5 (EVAg: 010V-04723; hCovN19/Netherlands/ZHNEMCN5892)
Omicron XBB.1.5 (GISAID: hCov-19/Netherlands/NH-EMC-5667)

Research involving human participants, their data, or biological material

Policy information about studies with human participants or human data. See also policy information about sex, gender (identity/presentation), and sexual orientation and race, ethnicity and racism.

Reporting on sex and gender	Sex or gender were not considered in study design. Sex was collected in the study design, and reported in the baseline characteristics table. A comparison of immunogenicity data from female and male participants is provided in Supplementary Figure S6.
Reporting on race, ethnicity, or other socially relevant groupings	Self-reported ancestry and hospital occupation were collected in the study design, and reported in the baseline characteristics table. No analyses based on these metadata were performed.
Population characteristics	Age and COVID-19 vaccination / infection history were the only covariates analyzed in this study.<br><br>Direct boost group (Omicron BA.1 bivalent booster vaccination):<br>(1) Ad26.COV2.S prime, BNT162b2 Omicron BA.1 boost, n = 43 (female:male, 29:14), median age 36 (range 22-44)<br>(2) Ad26.COV2.S prime, mRNA-1273.214 boost, n = 47 (female:male, 29:18), median age 53 (45-60)<br>(3) SARS-CoV-2 infection prime, BNT162b2 Omicron BA.1 boost, n = 6 (female:male, 3:3), median age 32 (range 27-44)<br>(4) SARS-CoV-2 infection prime, mRNA-1273.214 boost, n = 15 (female:male, 14:1), median age 52 (range 46-59)<br>(5) mRNA-based prime, BNT162b2 Omicron BA.1 boost, n = 40 (female:male, 31:9), median age 35 (range 18-44)<br>(6) mRNA-based prime, mRNA-1273.214 boost, n = 46 (female:male, 40:6), median age 53.5 (range 45-64)<br><br>Postponed boost group (Omicron BA.5 bivalent booster vaccination):<br>(7) Ad26.COV2.S prime, BNT162b2 Omicron BA.5 boost, n = 39 (female:male, 24:15), median age 47 (range 27-64)<br>(8) Ad26.COV2.S prime, mRNA-1273.222 boost, n = 41 (female:male, 30:11), median age 45 (range 25-59)<br>(9) SARS-CoV-2 infection prime, BNT162b2 Omicron BA.5 boost, n = 9 (female:male, 9:0), median age 50 (range 22-51)<br>(10) SARS-CoV-2 infection prime, mRNA-1273.222 boost, n = 9 (female:male, 8:1), median age 48 (range 28-62)<br>(11) mRNA-based prime, BNT162b2 Omicron BA.5 boost, n = 36 (female:male, 25:11), median age 46.5 (range 24-61)<br>(12) mRNA-based prime, mRNA-1273.222 boost, n = 33 (female:male, 23:10), median age 45 (range 21-61)
Recruitment	A total of 434 healthcare workers (HCW) were included in the SWITCH-ON trial after screening of 592 potential participants. Patients were recruited by public announcements (websites) at 4 different university hospitals in the Netherlands. No selection bias was present; however, participants reflect the Dutch HCW population (mostly female, white, between 18-65 years old).
Ethics oversight	The study protocol (MEC-2022-0462) was approved by the Medical Ethics Committee of Erasmus University Medical Center (Rotterdam, the Netherlands), the sponsor site, and the local review boards of the other participating centers at the Amsterdam University Medical Centers, the Leiden University Medical Center, and the University Medical Center Groningen. Written informed consent was obtained from all study participants prior to the first study visit. There was no incentive or compensation for participation in the study. The study is registered with ClinicalTrials.gov (NCT05471440).

Note that full information on the approval of the study protocol must also be provided in the manuscript.

Field-specific reporting

Please select the one below that is the best fit for your research. If you are not sure, read the appropriate sections before making your selection.

☒ Life sciences ☐ Behavioural & social sciences ☐ Ecological, evolutionary & environmental sciences

For a reference copy of the document with all sections, see nature.com/documents/nr-reporting-summary-flat.pdf

Life sciences study design

All studies must disclose on these points even when the disclosure is negative.

Sample size	A power calculation in the SWITCH-ON trial was performed to identify the number of participants required per study arm, namely: (i) Ad26.COV2.S prime in the DB group, (ii) mRNA-based prime in the DB group, (iii) Ad26.COV2.S prime in the PPB group and (iv) mRNA-based prime in the PPB group. For each arm, 91 participants were required to reach 80% power at a two-sided 5% significance level to detect a difference of 0.2 log10-transformed in the fold change of antibody response between vaccination day and 28 days after boost. This difference was based on the previous HCW study performed at Erasmus MC, in which the mean fold changes for adenovirus primed participants and mRNA-primed participants were reported as 1.344 (SD 0.451) and 1.151 (0.449), respectively.
Data exclusions	Participant exclusions are reported in Figure 1. Data was excluded if samples were not collected at the pre-specified timepoints after vaccination as described in the study protocol. Data availability is reported in Supplementary Table S4. Immune parameters after infection were analyzed separately.

Replication	All neutralization assays were measured in duplicate. Bridging samples were included in neutralization assays as internal controls, and assays were repeated when these bridging samples deviated significantly. All attempts at replication were successful.
Randomization	The SWITCH-ON trial comprised two groups to which the participants were randomly assigned: (1) a direct boost group (DB) (n=219) or (2) a postponed boost (PPB) group (n=183). Participants in the DB group were vaccinated in October 2022 with an Omicron BA.1 bivalent vaccine (BNT162b2 Omicron BA.1 or mRNA-1273.214, selection based on age, <45 yrs received Pfizer, ≥45 years received Moderna); participants in the PPB group were vaccinated in December 2022 with an Omicron BA.5 bivalent vaccine (BNT162b2 Omicron BA.5 or mRNA-1273.222, randomly assigned).
Blinding	Due to the set-up of the study, it was not possible to blind participants from randomization. Therefore, participants were informed about their group allocation prior to the first study visit. Randomization was completed by research assistants who were not involved in statistical analyses. Where necessary, sample selection was performed unblinded to allow equal sample numbers per subgroup. During data collection and analysis, researchers were blinded to sample information, and were only exposed to study IDs.

Reporting for specific materials, systems and methods

We require information from authors about some types of materials, experimental systems and methods used in many studies. Here, indicate whether each material, system or method listed is relevant to your study. If you are not sure if a list item applies to your research, read the appropriate section before selecting a response.

Materials & experimental systems

n/a	Involved in the study	
☐	☒	Antibodies
☐	☒	Eukaryotic cell lines
☒	☐	Palaeontology and archaeology
☒	☐	Animals and other organisms
☐	☒	Clinical data
☒	☐	Dual use research of concern
☒	☐	Plants

Methods

n/a	Involved in the study	
☒	☐	ChIP-seq
☒	☐	Flow cytometry
☒	☐	MRI-based neuroimaging

Antibodies

Antibodies used	rabbit anti-SARS-CoV-2 nucleocapsid antibody; Sino Biological, #40143-T62; polyclonal; dilution: 1:1,000<br>horseradish peroxidase (HRP)-labeled goat anti-rabbit IgG antibody; Dako, #P0448; polyclonal, dilution: 1:2,000<br>HRP-labeled rabbit anti-human IgG antibody; Dako, #P0214: polyclonal; dilution: 1:6,000
Validation	Sino Biological, the manufacturer of the rabbit anti-SARS-CoV-2 nucleocapsid antibody, validated the antibody for use in WB and ELISA applications, but not IHC/IF. Specificity of the antibody against SARS-CoV(-2) nucleocapsid protein was demonstrated by the manufacturer. Antibodies used in this study, including the rabbit anti-SARS-CoV-2 nucleocapsid antibody, were previously validated in-house. Antibodies were titrated and evaluated before the assays reported in this study were performed.

Eukaryotic cell lines

Policy information about cell lines and Sex and Gender in Research

Cell line source(s)	The human airway cell line Calu-3 was obtained through ATCC (#HTB-55).
Authentication	The cell line was not further authenticated.
Mycoplasma contamination	Cell lines were tested negative for mycoplasma contamination during regular screening procedures.
Commonly misidentified lines (See ICLAC register)	No commonly misidentified cell lines were used in this study.

Clinical data

Policy information about clinical studies

All manuscripts should comply with the ICMJE guidelines for publication of clinical research and a completed CONSORT checklist must be included with all submissions.

Clinical trial registration	NCT05471440
Study protocol	The Erasmus MC Medical Ethics Committee gave ethical approval for this work performed as part of the SWITCH study (MEC-2022-0462; NL81983.078.22). The protocol was submitted to the editor.
Data collection	The SWITCH-ON trial comprised two groups to which the participants were randomly assigned: (1) a direct boost group (DB) (n=219)

Data collection

or (2) a postponed boost (PPB) group (n=183). Participants in the DB group were vaccinated in October 2022 with an Omicron BA.1 bivalent vaccine (BNT162b2 Omicron BA.1 or mRNA-1273.214, selection based on age, <45 yrs received Pfizer, ≥45 years received Moderna); participants in the PPB group were vaccinated in December 2022 with an Omicron BA.5 bivalent vaccine (BNT162b2 Omicron BA.5 or mRNA-1273.222, randomly assigned). Samples were collected at 7 days, 28 days and 3 months post vaccination during working hours at the sponsor site (Erasmus University Medical Center) or the other participating university medical centers (Amsterdam University Medical Centers, the Leiden University Medical Center, and the University Medical Center Groningen), as pre-defined in the study protocol.

Outcomes

According to the study protocol, the primary outcome was the fold change (i.e., geometric mean ratio [GMR]) in antibody response between baseline and 28 days after boost across both groups. Secondary outcomes were fast response, S-specific T-cell response and levels of neutralizing antibodies. Here, we report observational data on magnitude and quality of the immunological response. Therefore, a descriptive approach was used to describe the immunogenicity of bivalent booster vaccinations over the period of 3 months following vaccination. We measured S-specific IgG binding antibodies, S-specific T-cell responses, and neutralization of the ancestral, BA.1, BA.5, and XBB.1.5 variants. Similar parameters were analyzed in the infection sub-study.

Plants

Seed stocks

N/A

Novel plant genotypes

N/A

Authentication

N/A
